# Benign fibrous histiocytoma of the bladder

**DOI:** 10.4103/0970-1591.30273

**Published:** 2007

**Authors:** Michelle De Padua, N. Subramanium

**Affiliations:** Department of Pathology, Apollo Hospitals, Hyderabad, India; * Department of Urology, Indraprastha Apollo Hospitals, New Delhi, India

**Keywords:** Benign, bladder, fibrous, histiocytoma

## Abstract

Mesenchymal tumors of the bladder are rare with leiomyoma accounting for most of these. We present a rare case of a bladder benign fibrous histiocytoma in a 52-year-old male. He presented with history of straining during micturition since two years. The magnetic resonance imaging revealed a large intravesical mass. The mass was excised. It weighed 600g, with a maximum dimension of 13cm. Histology was that of a benign fibrous histiocytoma. To our knowledge, only two cases of this tumor have been reported in the bladder so far. The clinical and pathological features are discussed.

## INTRODUCTION

Mesenchymal tumors of the bladder are rare with leiomyomas accounting for most of the cases.[1] Fibrous histiocytoma is a benign tumor usually occurring in the dermis and superficial subcutis. It is also uncommonly found in deep soft tissue and sporadically in parenchymal organs. Only two cases of this tumor in the bladder have been so far reported in the literature.[2,3] We present the third case.

## CASE REPORT

**Figure 1:**
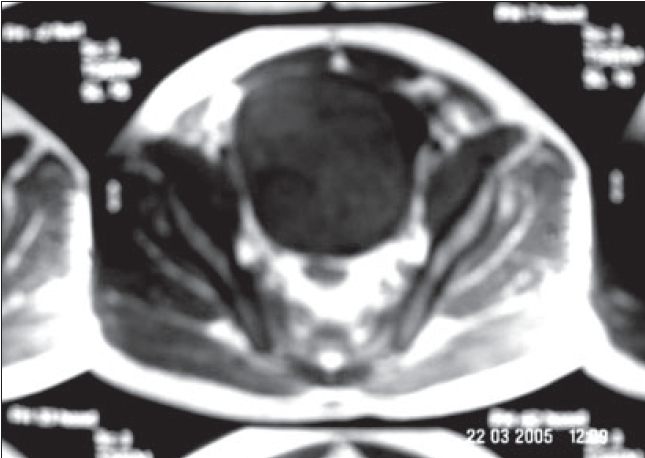
MRI- Well circumscribed intravesical mass

A 52-year-old male presented with complaints of straining during micturition for two years, with poor stream of urine and burning micturition on and off. There was no history of hematuria, pyuria or retention of urine. Systemic systems were not present. On examination, suprapubic lump was palpable, more on the right side. On cystoscopy, a large solid tumor arising from just above the bladder neck along the right lateral wall, going anteriorly was seen. The magnetic resonance imaging (MRI) revealed a large encapsulated intravesical mass [Figure 1]. There was no obvious extravesical extension. The radiological features were suggestive of a benign etiology, which could represent a mesenchymal tumor. Needle biopsy was performed followed by excision of the tumor. During surgery, an infraumbilical midline incision was taken, with an extraperitoneal approach. The tumor was palpated and bladder opened just lateral to the tumor on the anterior surface. Mucosa over the tumor was incised and a plane was developed. The tumor was freed from all mucosal attachments and enucleated. Pedicle of the tumor was transfixed and tumor was delivered.

### Pathological findings

**Figure 2:**
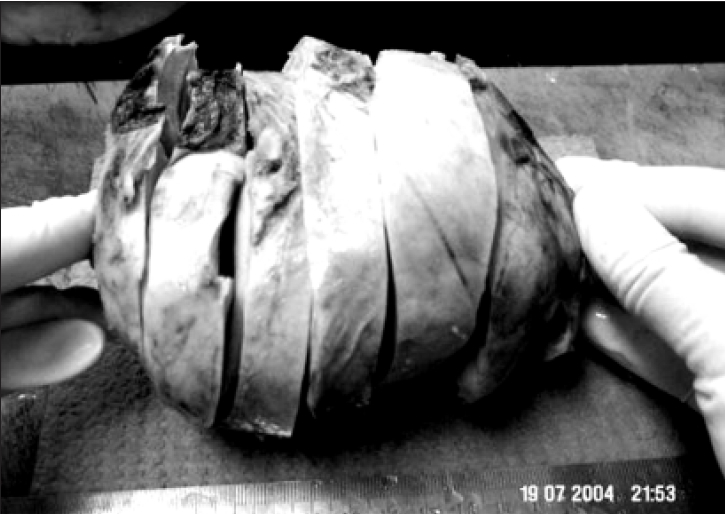
Gross: Encapsulated mass weighing 600 gms and measuring 13 × 10 × 9 cms

**Figure 3:**
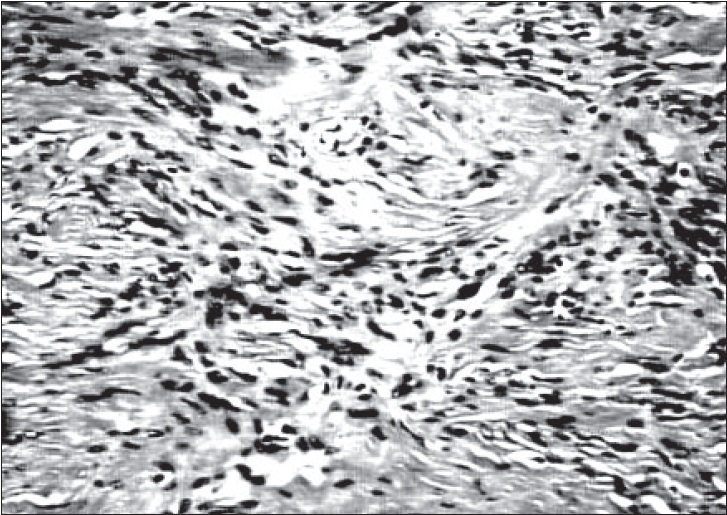
Spindle cells arranged in a storiform pattern (H/E, 20×)

**Figure 4:**
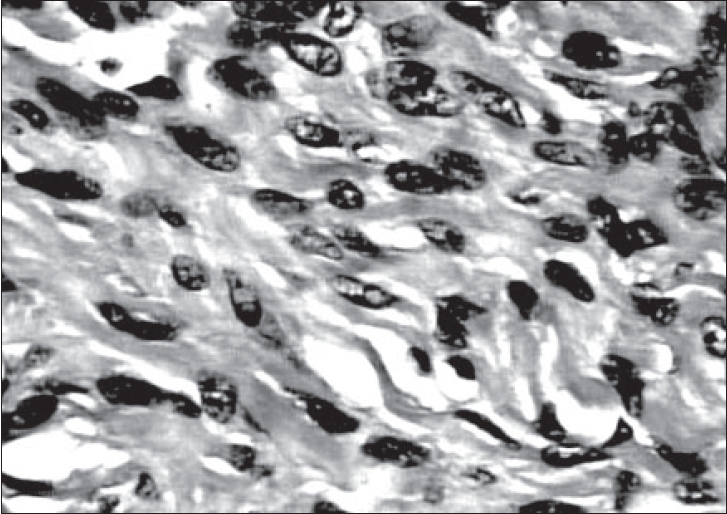
Spindle cells with uniform nuclei (H/E, 40×)

Histology of the biopsy revealed a neoplasm composed of neoplastic cells with uniform oval to spindled nuclei arranged in a striking storiform pattern [Figure 2]. There was no evidence of necrosis, mitotic activity or nuclear pleomorphism. Immunohistochemical stain with vimentin was strongly positive. Cytokeratin, smooth muscle actin, S-100 and desmin was negative. A diagnosis of a mesenchymal tumor, possibly benign was offered at this stage, since th amount of tissue studied was small. Subsequently, the excised specimen was received. Grossly, the tumor measured 13 × 10 × 9 cm and weighed 600g [Figure 3]. It was well circumscribed with a smooth surface. Cut surface showed a firm, nodular, yellowish white appearance. Histology revealed features as seen in the needle biopsy specimen. There was no evidence of nuclear atypia or mitotic activity in multiple sections studied [Figure 4]. The final diagnosis was benign fibrous histiocytoma.

## DISCUSSION

Mesenchymal tumors of the bladder are rare with leiomyomas accounting for most of the cases.[1] Other benign mesenchymal tumors of the bladder include hemangioma, lymphangioma, neurofibroma, neurilemmoma, granular cell tumor, benign fibrous histiocytoma and lipoma.[1] In tumors of fibrohistiocytic origin of the bladder, malignant fibrous histiocytoma has been reported more often than benign fibrous histiocytoma.[4–9] Only two cases of benign fibrous histiocytoma of the bladder have been reported so far.[2,3] The unusual feature in our case is its large size (13 cm). Fibrous histiocytoma is a benign tumor usually occurring in the dermis and superficial subcutis. It is also uncommonly found in deep soft tissue and sporadically in parenchymal organs. These tumors are clinically benign and complete excision is usually curative. While degerative atypia, necrosis, some level of mitotic activity and increased cellularity may be seen as an isolated finding, increased mitotic activity with nuclear atypia should raise concerns about malignancy.[10] There was no evidence of nuclear atypia, necrosis or mitotic activity in our case. Complete surgical excision was achieved in our patient. There is no evidence of recurrence after 10 months of follow-up. The most controversial aspect of this tumor is its histogenesis. It has been variably interpreted as a histiocytic and fibroblastic tumor. The presence of lysozymes and proteolytic enzymes has been interpreted as evidence favoring a histiocytic origin.[11] The predominant fibroblastic appearance of the cells ultrastructurally and lack of histiocytic markers have been used in support of a fibroblastic origin.[12] Immunohistochemistry helps in differentiating from other tumors like leiomyoma, neurofibroma, schwannoma etc.

## References

[1] Eble JN, Young RH (2000). Diagnostic Histopathology of tumours. Christofer DM Fletcher.

[2] Stearns MM, Mitchell AD, Powell NE, Wood WG, Mebust WK (1976). Fibrous histiocytoma of the bladder. J Urol.

[3] Karol JB, Eason AA, Tanagho EA (1977). Fibrous histiocytoma of bladder. Urology.

[4] Lema Grille J, Rodriguez Nunez H, Cimadevila Garcia A, Durana Tonder C, Blanco Parra M (2001). Malignant vesico-prostatic fibrous histiocytoma. Actas Urol Esp.

[5] Anderson JD, Scardino P, Smith RB (1977). Inflammatory fibrous histiocytoma presenting as a renal pelvic and bladder mass. J Urol.

[6] McCormick SR, Dodds PR, Kraus PA, Lowell DM (1985). Nonepithelial neoplasms arising within vesical diverticula. Urology.

[7] Henriksen OB, Mogensen P, Engelholm AJ (1982). Inflammatory fibrous histiocytoma of the urinary bladder: Clinicopathological report of a case. Acta Pathol Microbiol Immunol Scand (A).

[8] Okuno T, Masuda M, Yamazaki A, Hirokawa M, Mat sushita K, Asakura S (1991). Malignant fibrous histiocytoma of the urinary bladder: A case report. Nippon Hinyokika Gakkai Zasshi.

[9] Oesterling JE, Epstein JI, Brendler CB (1990). Myxoid malignant fibrous histiocytoma of the bladder. Cancer.

[10] Fletcher CD (1990). Benign fibrous histiocytoma of subcutaneous and deep soft tissue: A clinicopathologic analysis of 21 cases. Am J Surg Pathol.

[11] du Boulay CE (1982). Demonstration of alpha-1-antitrypsin and alpha-1-anti-chymotrypsin in fibrous histiocytomas using immunoperoxidase technique. Am J Surg Pathol.

[12] Kamino H, Salcedo E (1999). Histopathologic and immunohistochemical diagnosis of benign and malignant fibrous and fibrohistiocytic tumours of the skin. Dermatol Clin.

